# Analysis of Velocity, Power and Skin Temperature in Paralympic Powerlifting Athletes with Fixed and Variable Resistance

**DOI:** 10.3390/sports12090250

**Published:** 2024-09-11

**Authors:** Gildo Francisco dos Santos Filho, Felipe J. Aidar, Márcio Getirana-Mota, Ciro José Brito, Esteban Aedo-Muñoz, Ângelo de Almeida Paz, Joilson Alves de Souza Leite Júnior, Edson Lucas Monteiro Vieira, Pantelis T. Nikolaidis

**Affiliations:** 1 Graduate Program of Physical Education, Federal University of Sergipe, São Cristóvão 49100-000, Brazil; gildofrancisco@hotmail.com (G.F.d.S.F.); fjaidar@gmail.com (F.J.A.); marcio_getirana@hotmail.com (M.G.-M.); angelo-paz@outlook.com (Â.d.A.P.); joilsonasljunior@gmail.com (J.A.d.S.L.J.); edsonn.edf@gmail.com (E.L.M.V.); 2 Group of Studies and Research of Performance, Sport, Health and Paralympic Sports—GPEPS, The Federal University of Sergipe, São Cristóvão 49100-000, Brazil; 3 Graduate Program of Physiological Science, Federal University of Sergipe, São Cristóvão 49100-000, Brazil; 4 Department of Physical Education, Federal University of Juiz de Fora, Governador Valadares 35010-180, Brazil; ciro.brito@ufjf.br; 5 Departamento de Educación Física, Deportes y Recreación, Universidad Metropolitana de Ciencias de la Educación, Santiago 7760197, Chile; esteban.aedo@usach.cl; 6 School of Health and Caring Sciences, University of West Attica, Egaleo, 12243 Athens, Greece

**Keywords:** paralympic powerlifting, variable resistance, chains, muscle strength, muscle fatigue

## Abstract

Variable resistance training has been widely used in athletic preparation. Objectives: To analyze the use of currents (VRT) and the traditional method (TRAD) on speed, power and temperature in a training session. Methods: Fourteen paralympic powerlifting (PP) athletes took part over three weeks. In week 1, familiarization and 1RM tests took place, and, in weeks 2 and 3, pre- and post-training took place, where the propulsive mean velocity and power and temperatures were assessed before and after, at 24 h and 48 h. Results: There was a difference in the sternal pectoral temperatures before and after VRT (*p* = 0.040) and at 48 h for TRAD and VRT (*p* = 0.018); in the clavicular pectoralis before and after VRT and TRAD (*p* = 0.003); in the anterior deltoid after and at 48 h for TRAD and VRT (*p* = 0.026 and *p* = 0.017); and in the triceps after and at 24 h and 48 h between TRAD and VRT (*p* = 0.005). In the training series, the MPV was significant in TRAD between Set1 and Set5 (*p* = 0.003), in training (VRT) between Set1 and Set5 (*p* = 0.001) and in Set5 between the methods (*p* = 0.047). For power, there was a difference between Set1 and 5 in TRAD (*p* = 0.016) and VRT (*p* = 0.002). Conclusion: We conclude that training with currents (VRT) promoted greater muscle fatigue when compared to traditional training.

## 1. Introduction

Dynamic training with constant external loads (isoinertial or traditional) is the most common method for strength preparation in athletic training [1]. However, variable resistance training (VRT) methods have gained popularity due to evidence suggesting that constant loads may not provide optimal resistance throughout the entire range of motion [2]. Studies have shown that the barbell decelerates in the final portion of the concentric phase, and the force production in the bench press is lower in the “sticking region” and the final portion of the movement [3,4].

VRT, on the other hand, offers a training modality that could address these issues. Exercises with an ascending force curve, such as the bench press, tend to benefit from VRT as it provides lower resistance in the “sticking region” and greater resistance near the end of the concentric phase, thereby enhancing force and velocity production [5]. Additionally, VRT has been observed to yield superior gains in maximal strength, velocity, and power [6,7]. Thus, it appears that training with variable resistance, with the use of chains and elastic bands, would offer resistance at points that traditional training does not, causing differentiated neuromuscular responses [8,9,10]. However, all studies included in these reviews were conducted on non-disabled individuals. Only one study has investigated VRT in athletes with lower limb disabilities, specifically in paralympic powerlifting (PP) athletes. Aidar et al. [8] found that the rate of force development after a VRT intervention was significantly lower compared to traditional training, indicating a higher effort demand for VRT conditions.

It is also known that the velocity profile for PP athletes differs from that of non-disabled individuals [11,12], and these athletes are generally stronger than conventional powerlifting athletes [13]. There is a growing body of research on this population, focusing on the impact of injuries on strength [14], training methods [8,11,15] and recovery methods [16,17,18], among others. Given the previous discussions, it is necessary to investigate the use of VRT in the PP population, as the only study conducted in this population examined only static strength variables. Furthermore, the literature suggests that VRT can acutely increase both the velocity and power in these athletes. Regarding the skin temperature and its measurement in relation to the type of training, intensity, duration, the muscles involved, and subcutaneous fat, the response of the muscle temperature during exercise after and up to 48 h later was observed in response to the type and intensity of the exercise. Thus, the temperature of the muscles involved would increase during high-intensity anaerobic training, decrease after training and increase in the following days, being an important marker of the intensity, in addition to being able to be used as a training control [19,20,21]

Therefore, our objectives were (1) to analyze the effects of traditional training and current training on the skin temperature (pectoralis major, deltoid and triceps brachii on the dominant side) before, after and at 24 and 48 h after training and (2) to evaluate the effect of traditional training and current training on dynamic indicators of strength (speed and power) during training with a load of 80% of 1RM and to evaluate speed and power with a load of 45% of 1RM before, after and at 24 and 48 h after a training session in the two training conditions mentioned in paralympic powerlifting. We hypothesized that the dynamic indicators of strength, in training with current training, would present lower values and higher skin temperatures than in traditional training.

## 2. Materials and Methods

### 2.1. Design

This study used a randomized, cross-over, counterbalanced design to analyze the effects of two different training methods (traditional and variable resistance using chains attached to the ends of the bar) on the performance of paralympic weightlifting (PP) athletes through dynamic strength tests. This was a quadruple-experimental study with a convenience sample. Participants were randomized to two experimental conditions: traditional training and training using chains. The study duration was three weeks. All tests were conducted on separate days, at the same time (between 9:00 AM and 12:00 PM), at temperatures ranging from 23 to 25 °C, with relative humidity of ~60%. All tests were performed using an adapted flat bench press.

In the first week, a familiarization session with the chain and traditional (invariable resistance) training methods was conducted, and a one-repetition-maximum (1RM) test was performed to determine the training load. In the second and third weeks, athletes were randomly assigned to the two training types: chain-based and traditional. Thus, the sample was equally divided with the same number of participants (50%) in each week.

**Figure 1:**
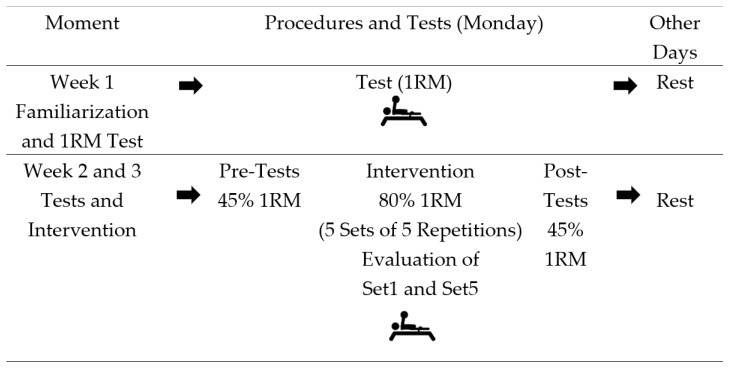
Study design.

During weeks 2 and 3, the dynamic force indicators (mean propulsive velocity and power) and skin temperature were evaluated at four time points: before, immediately after, 24 h after and 48 h after the intervention. The intervention consisted of five sets of five repetitions (5 × 5) at 80% of 1RM. During the intervention, the dynamic force indicators were assessed in the first (Set1) and last series (Set5), as illustrated in Figure 1.

### 2.2. Sample

This study included 14 paralympic powerlifting (PP) athletes who competed at the national level and met the criteria established by the World Para Powerlifting Organization [22]. The participants had various disabilities: five had malformations (arthrogryposis), three had spinal cord injuries below the eighth vertebra due to trauma, three had sequelae of poliomyelitis and three had amputations (Table 1).

The inclusion criteria required all athletes to have a minimum of 18 months of training experience and prior participation in national-level competitions. These athletes were ranked among the top 10 in the country. The exclusion criteria included pain or an inability to perform the tests or voluntary withdrawal from the study.

The sampling power was calculated a priori using the open software G*Power version 3.1.9.6 (University of Kiel, Germany), with the “F family (ANOVA)” considering a standard f = 0.8, α < 0.05, 1 − β = 0.80. In this way, it was possible to estimate the sample power for a minimum sample of twelve subjects, suggesting that the study had statistical strength to respond to the research objective, as this was associated with other studies that adopted a similar sample

All athletes voluntarily participated in this study and provided written informed consent in accordance with Resolution 466/2012 of the National Research Ethics Commission (CONEP) of the National Health Council and following the ethical principles of the Declaration of Helsinki (1964, revised in 2013) of the World Medical Association [2,3]. This study was approved by the Research Ethics Committee of the Federal University of Sergipe (ID-CAAE: 67953622.7.0000.5546), technical opinion number 6.523.247, dated 22 November 2023.

### 2.3. Instruments and Procedures

For athlete weighing, a specialized electronic digital scale for wheelchair individuals was used (Model Mic Welcair; Michetti, São Paulo, Brazil). The scale dimensions were 1.50 × 1.50 m with a maximum capacity of 300 kg. The bench press exercise was performed on an official 210 cm flat bench with a 220 cm bar and weights, all manufactured by Eleiko (Sweden) and approved by the International Paralympic Committee (IPC) [22].

The chains used for the variable resistance overload measured between 1.00 and 1.50 m. The chains were added to the training loads, representing 20% of the total load. Thus, in traditional training, the load was 80% of 1RM exclusively with weights. In chain training, 80% was weights and 20% was chains. For example, if an athlete could lift 100 kg in the bench press for a single repetition (1RM), the traditional training load would be 80 kg with weights only. In chain-based training, 64 kg of weights and 16 kg of chains were used. The chain weight was determined using the mean chain distance and measured with a digital dynamometer (Data Weighing Systems, Wood Dale, IL, USA) with a maximum weight of 150 kg and resolution of 0.05 kg. The chains weighed 7.0, 3.5 and 3.0 kg.

The training session consisted of five sets of five repetitions (5 × 5) [8], with a full range of motion. The difference between the training sessions was the use of chains attached to the bar (variable resistance) or fixed resistance (invariable resistance). All athletes had prior experience with both chain-based and fixed resistance training.

In weeks 2 and 3, prior to the interventions, the athletes performed a warm-up targeting the main muscle groups involved in upper limb movement. Three exercises were used (shoulder abduction with dumbbells, elbow extension on a pulley and shoulder rotation with dumbbells), with three sets of 10 to 20 RM for approximately 10 min. Subsequently, a specific warm-up was performed on the flat bench press, with 30% of the 1RM load, consisting of 10 slow and 10 fast repetitions. During testing, the athletes were verbally encouraged to perform their best.

During the interventions, the athletes performed 5 sets of 5 repetitions (5 × 5), with chains fixed to the bar or fixed resistance (weights only). Assessments were conducted before, immediately after, 24 h after and 48 h after the training session. Dynamic tests at 45% of 1RM were performed using a valid and reliable linear position transducer [24], measuring the mean propulsive velocity (MPV) and power (P). The body temperature was measured using an infrared thermographic camera before, immediately after, 24 h after and 48 h after the training session.

### 2.4. Maximum Load Test (1RM)

A one-repetition-maximum (1RM) test was conducted to establish the training load for both the traditional and variable resistance training (VRT) methods. This test was performed in week 1. Each participant selected an initial load that they believed they could lift, and incremental weights were added until their maximum lift capacity was reached. Whenever an athlete failed to lift the load, the weight was reduced by 2.4% to 2.5%. Participants rested for 3 to 5 min between attempts. The 1RM test was conducted 72 h prior to the training sessions [8].

### 2.5. Dynamic Force Measurements

**Figure 2:**
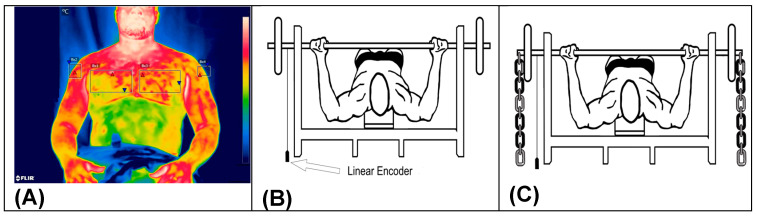
Infrared thermography photo model (**A**), detail of the placement of the linear encoder connected to the bar (**B**) and representation of the placement of the chains for training with variable resistance (**C**).

A linear position transducer was vertically attached to the left side of the barbell using a Velcro strap. To measure the mean propulsive velocity (MPV) and power (POW), a linear encoder from the Speed4Lift measurement system (Vitruve, Madrid, Spain) [24] was used to quantify the vertical displacement velocity, connected to the bench press bar. These parameters were analyzed before, immediately after, 24 h after and 48 h after the training session using a load of 45% of 1RM, where the velocity was expected to be approximately 1.0 m·s^−1^ [4]. The velocity data for the concentric phase were considered to begin at the ascending component of the movement and terminate at full elbow extension. The MPV and power were collected for analysis with loads of 45% of 1RM at the aforementioned time points, as well as during the first and last sets of the intervention with loads of 80% of 1RM (Figure 2).

### 2.6. Skin Temperature Measurement

The acquisition of thermal images was conducted in a specially prepared room without natural light and with a black background. The room had no directed airflow towards the collection area, and the ambient temperature was controlled by an air conditioning unit, maintained at approximately 25 °C, with relative humidity between 55% and 60%. The environmental temperature conditions were monitored using a Hikari HTH-240 thermo-hygrometer (Hikari, Shenzhen, China) [20,25]. These measurements were performed according the recommendations of Themographic Imaging in Sports and Exercise Medicine.

Participants were instructed to refrain from vigorous physical activity for 24 h prior to the assessment and to avoid applying any creams or lotions to their skin in the 6 h immediately preceding the evaluation. To obtain the thermograms, each athlete remained seated and was instructed to avoid sudden movements, not to cross their arms and to refrain from scratching for an acclimatization period of at least 10 min.

Images were captured using a FLIR T640sc infrared camera (Flir, Stockholm, Sweden) with a measurement range of −40 °C to 2000 °C, accuracy of 2%, sensitivity < 0.035, an infrared spectral band of 7.5–14 μm, a refresh rate of 30 Hz and a resolution of 640 × 480 pixels. Thermal image analysis was performed using the FLIR TOOLS software (Flir, Stockholm, Sweden). The regions of interest evaluated were the anterior and posterior aspects of the trunk and arms [18,26] (Figure 2A).

### 2.7. Overload with the Use of Chains

The participants were positioned supine on an Eleiko Paralympic bench press (Eleiko, Halmstad, Sweden). They held the barbell in two positions: with the elbows fully extended and with the bar in contact with the chest. This distance was measured to ensure that only the resistance produced by the chains was quantified. Subsequently, the athletes replaced the bar on the supports, and the resistance produced solely by the chains was measured in both the upper and lower positions. The mean resistance of the chains throughout the entire range of motion was set as close as possible to 20% of the working load [27]. The athletes performed training sessions consisting of five sets of five repetitions maximum (5 × 5 at 80% 1RM) (80% = 20% 1RM chain + barbell weight) with chains (variable resistance) attached to the ends of the barbell. The load from the chains was quantified to be as close as possible to 20% of 1RM throughout the range of motion [28] (Figure 2C).

### 2.8. Statistics

Central tendency measures, including the mean and standard deviation (X ± SD), as well as the 95% confidence interval (CI 95%), were utilized. Given the sample size, the Shapiro–Wilk test was conducted to verify the normality of the data. A two-way ANOVA (moment × training) for repeated measures, followed by Bonferroni post hoc tests, was employed. The effect size was assessed using the partial eta squared (η^2^_p_), with magnitudes classified as low < 0.05, medium 0.05 to <0.25, high 0.25 to <0.50 and very high ≥0.50 [29,30]. Statistical analysis was performed using the Statistical Package for Social Sciences (SPSS), version 24. A significance level of *p* < 0.05 was adopted for all analyses (IBM, New York, NY, USA).

## 3. Results

Table 2 shows the results in relation to the skin temperature before, after and 24 and 48 h later, under traditional training conditions and with the use of currents.

There was a significant difference in the skin temperature over active muscles at different times in the sternal pectoralis during training with currents before (33.36 ± 1.45) and after (34.64 ± 1.95) (*p* = 0.040, F = 2.212, η^2^_p_ = 0.145) and also at 48 h (33.53 ± 1.34) using the traditional method and at 48 h for the currents (34.36 ± 1.69) (*p* = 0.018, F = 11.966, η^2^_p_ = 0.479). For the clavicular pectoral, there was a difference in the temperature before (33.79 ± 1.31) and after (35.79 ± 3.68) training using currents (*p* = 0.003, F = 4.495, η^2^_p_ = 0.257). In relation to the anterior deltoid muscle, the differences were later (34.18 ± 1.46) in traditional training and then with chains (36.21 ± 3.51) (*p* = 0.026, F = 17.181, η^2^_p_ = 0.257) and also at the traditional 48 h time (33.68 ± 1.00) and 48 h with currents (34.50 ± 1.61) (*p* = 0.017, F = 17.181, η^2^_p_ = 0.569). For the triceps, there was a difference in the temperature in the moments after the traditional method (32.81 ± 1.23) and with currents (33.71 ± 1.54), at 24 h for traditional training (32.84 ± 1.65) and with currents (33.50 ± 1.61) and at 48 h for traditional training (32.26 ± 1.09) and with currents (33.00 ± 1.18) (*p* = 0.005, F= 17.781, η^2^_p_ = 0.578).

**Figure 3:**
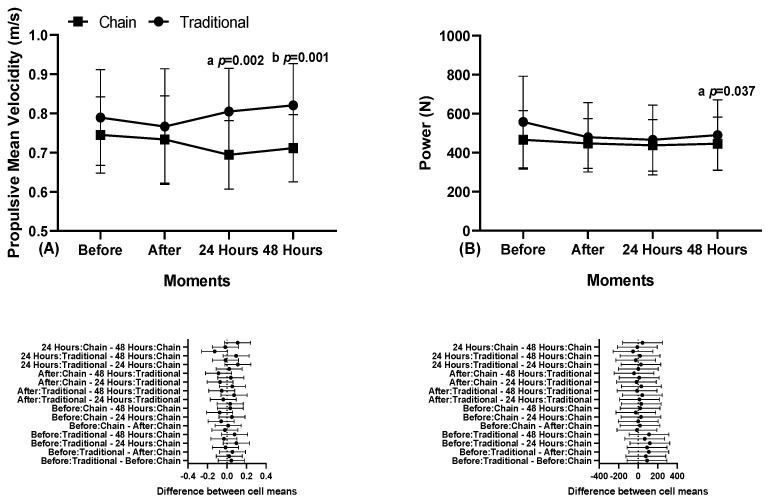
Mean propulsive velocity (**A**) and power (**B**), with 45% of 1RM, at different times, in traditional training conditions and with variable resistance (use of chains).

Figure 3 shows the results of the mean propulsive velocity and power with 45% of 1RM, evaluated at different times with traditional training and the use of chains.

Regarding the mean propulsive velocity with 45% of the training load, the result shows that there was a difference 24 h later in the mean propulsive velocity (MPV) between the traditional training conditions (0.80 ± 0.11 m/s, CI 95% 0.74–0.87) and with currents (0.69 ± 0.09 m/s, 95% CI 0.64–0.74, “a” *p* = 0.002) and there was also a difference at 48 h between traditional training (0.82 ± 0.11 m/s, 95% CI 0.76–0.88) and with currents (0.71 ± 0.09, 95% CI 0.66–0.76, “b” *p* = 0.001, F = 13.826, η^2^_p_ = 0.567). Regarding the power, there was a difference in the moment 48 h later between traditional training (490.54 ± 180.73 W, 95% CI 386.19–594.89) and with currents (446.43 ± 136.26 W, CI 95% 367.75–526.10, “a” *p* = 0.037, F = 4.686, η^2^_p_ = 0.294).

**Figure 4:**
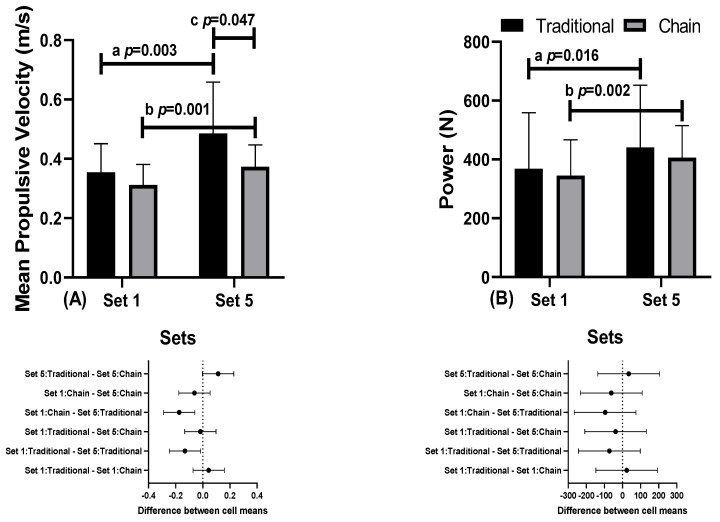
Mean propulsive velocity (**A**) and power (**B**), with 80% of 1RM, during the training session with 80% of 1RM, in the first series (Set1) and in the last series (Set5), in traditional training conditions and with variable resistance (use of chains).

Figure 4 shows the results of the mean propulsive velocity and power with 80% of 1RM, evaluated in the first series (Set1) of the training session and in the last series (Set5), with five series of five repetitions performed in the traditional training conditions and training with the use of chains.

During the training session with 80% 1RM, the MPV and power were evaluated in the first series (Set1) and in the last series (Set5). There was a significant difference in the MPV in traditional training between Set1 (0.35 ± 0.10 m/s, 95% CI 0.30–0.41) and Set5 (0.49 ± 0.17 m/s, 95% CI 0.39–0.59, “a” *p* = 0.003, F = 21.255, n^2^_p_ = 0.510) and between Set1 (0.31 ± 0.07, 95% CI 0.27–0.33) and Set5 (0.37 ± 0.07, 95% CI 0.33–0.42, “b” *p* = 0.001, F = 4.166, η^2^_p_ = 0.556). There were differences in Set1 between the traditional training conditions (0.35 ± 0.10 m/s, CI 95% 0.30–0.41) and training with currents (0.31 ± 0.07, CI 95% 0.27–0.33, *p*= 0.047, F = 3.854, η^2^_p_ = 0.270).

Regarding the power, there was a difference in traditional training between Set1 (368.45 ± 190.64 W, 95% CI 258.38–478.53) and Set5 (440.73 ± 211.79 W, 95% CI 318.45–563.02, “a” *p* = 0.016, F = 21.147, η^2^_p_ = 0.371). There was a difference in training with the use of currents between Set1 (344.84 ± 122.16 W, 95% CI 274.31–415.37) and Set5 (406.59 ± 108.55 W, 95% CI 343.92–469.26, “b” *p* = 0.002, F = 21.147, η^2^_p_ = 0.527).

## 4. Discussion

The primary objective of this study was to evaluate the muscle temperature, bar velocity profile and power in paralympic powerlifting athletes. Our results indicated temperature differences between the two training conditions (traditional vs. chain-based) and within the chain-based condition itself.

As shown in Table 2, there were significant temperature differences between the conditions post-intervention (anterior deltoid and triceps brachii), at 24 h (triceps brachii) and especially at 48 h post-intervention (sternal pectoralis, anterior deltoid and triceps brachii). It is well documented that there are differences in electrical activity among these muscles during the bench press [31], particularly in this specific population [32,33]. Strength training has been observed to induce changes in skin temperature [20], and thermography can be a valuable tool for the monitoring of training loads and identification of injury risks [21,34].

Our study demonstrated that training with chains resulted in a greater increase in temperature compared to traditional training. According to Garagiola and Giani [35], increased blood flow variations can lead to local temperature increases (hyperthermia). Elevated skin temperature profiles may be associated with increased blood flow due to a potential inflammatory response [36]. In our study, at 48 h post-intervention, temperature differences between the conditions were still evident: 0.83 °C for the sternal pectoralis, 0.82 °C for the anterior deltoid and 0.74 °C for the triceps. Marins et al. [37] classified the temperature differences into five levels: normal (<0.4 °C), follow-up (0.5–0.7 °C), prevention (0.8–1 °C), alarm (1.1–1.5 °C) and severity (>1.6 °C). According to these classifications, at 48 h post-intervention, the chain-based training condition would be classified as “prevention” for the sternal pectoralis and anterior deltoid and “follow-up” for the triceps. This indicates that the chain-based training imposed a greater load on the sternal pectoralis and anterior deltoid (Table 1).

Anecdotal evidence suggests that the use of chains increases the perceived effort for practitioners [38]. This may be substantiated by our findings, as the muscle temperatures of the sternal pectoralis, anterior deltoid and triceps brachii remained elevated at 48 h post-intervention compared to the traditional method. Elevated temperatures for a period equal to or greater than 24 h indicate muscle damage [39], demonstrating that chain-based training induced greater physical effort than traditional training with the same absolute loads.

Regarding the mean propulsive velocity, as shown in Figure 1 and Figure 2, the velocity profile was lower for the chain-based condition. There are conflicting data on the velocity profile with the addition of chains to the barbell. Coker et al. [38] found that the bar velocity was lower with chains compared to without chains (5% of the load constituted by chains). Conversely, Baker and Newton [40] found that adding chains increased the bar velocity compared to without chains (12–16% of the load constituted by chains). However, neither study found significant differences between the velocities of the two conditions, with only differences in the absolute values.

A recent meta-analysis by Shi et al. [7] identified that variable resistance training is more effective in acutely increasing the velocity and power than constant load training. However, our data do not corroborate this, possibly due to sensitivity to the constant load and total volume between repetitions and sets required to generate higher acute velocities. Our study used 80% of 1RM (60% constant load/20% variable resistance in a five-set, five-repetition protocol), whereas the studies included in the cited review used loads below or above 80% of 1RM, with a maximum of three sets and six repetitions per set. It is known that a longer time under tension results in greater oxidative stress and muscle fatigue [17], which may partially explain the discrepancy in the results.

The bench press is an exercise with an ascending force curve, meaning that the maximum force and power production capacity is near full elbow extension [41]. This suggests that sensitivity to the total load and volume may be necessary for variable resistance to acutely increase the execution velocity in the bench press. When the bar touches the sternum, it is a moment of mechanical disadvantage, and, if the total resistance at this angle does not promote greater acceleration, the individual will not achieve higher velocities even with variable resistance.

Regarding the potential sensitivity to the total load and volume for a higher velocity using variable resistance, it may result from preparatory muscle stiffness, where the individual generates a neural signal. Consequently, when completing the eccentric phase of the bench press, the additional weight from variable resistance (rubber or chain) is not active at the initial concentric phase, generating post-activation potentiation during each movement [40]. However, this condition was not observed in our study, suggesting sensitivity to the total load and volume used in lifting. Our study used a total load of 80% of 1RM with five sets of five repetitions, while the studies analyzed by Shi et al. [7] in their meta-analysis used total loads below or above 80% of 1RM but with lower volumes.

Supporting our hypothesis, van den Tillaar et al. [42] conducted an experiment using 85% of 1RM with a constant load and 5.1% load using chains in two conditions: (1) 5.1% added in the final phase of the eccentric execution and (2) 5.1% added in the final phase of the concentric contraction. They found no differences for the constant load condition compared to condition 1 and observed significant negative differences in the velocity for condition 2 compared to a constant load, aligning with our results.

Additionally, when analyzing loads of 45% of 1RM (Figure 2), chain-based training showed significant negative differences at 48 h post-intervention compared to constant load training. Loads representing 45% of 1RM are considered easy or light [11,43], indicating that constant load training tends to have faster recovery than chain-based training.

Regarding the power, our findings also revealed lower power production in the chain-based condition compared to the constant load (Figure 1 and Figure 2). As expected, since the power is a product of the force and velocity, if either variable (force or velocity) does not reach its maximum, the power will be negatively affected. Unlike our study, Godwin et al. [44] did not find higher power production with chains compared to a constant load, despite finding significant positive differences for the chain condition. Although the power production was higher with chains, these values did not significantly differ from those in the constant load condition.

Another interesting finding is that the velocity profile with 80% of 1RM was always lower in the first set compared to the last, regardless of the condition analyzed. Getirana-Mota et al. [9] also identified this condition in their study, suggesting that it may result from the possible bodily adjustment to the bench, especially since our sample consisted of athletes with physical disabilities, potentially influencing the velocity profile of the first set.

Some limitations were observed in the study. Our sport has a single classification, with athletes being eligible or not for the sport, which could present different outcomes for different disabilities. We did not separate the athletes into body weight categories, since athletes in lighter categories have greater relative strength and heavier athletes have greater absolute strength. On the other hand, other forms of variable resistance could present different results, such as the use of rubber. Another limitation is due to the sample size; despite being a sample of national- and international-level athletes, the number of athletes who participated in the study was limited. Moreover, future research could investigate other forms of variable resistance and different overloads, since our study used only an overload with chains and with a fixed value in terms of the percentage for this implement, since these variations could impact the performance.

## 5. Conclusions

Based on the analysis and discussion of the data, we conclude that variable resistance training (VRT) induces greater neuromuscular fatigue, persisting even 48 h after the training session. The thermographic analysis revealed that the sternal portion of the pectoralis major and the anterior deltoid muscles were the most overloaded during VRT. Furthermore, when examining the velocity and power profiles, these were consistently inferior to those in traditional training.

Our initial hypothesis posited that the muscle temperatures would return to pre-intervention levels after 24 h; however, this was not observed. We also hypothesized that the velocity and power profiles would be higher for the VRT condition. Contrary to our expectations, in none of the analyzed situations were higher velocities or power production observed with VRT, even when the load was 45% of 1RM.

These findings suggest that VRT may impose a greater physiological demand on paralympic powerlifting athletes compared to traditional training, potentially necessitating longer recovery periods. The persistent elevation in muscle temperature and reduced performance in terms of the velocity and power metrics indicate that coaches and athletes should carefully consider the implementation and periodization of VRT in training programs for this population.

Future research should investigate the long-term adaptations to VRT in paralympic powerlifting athletes and explore optimal loading strategies that may mitigate the acute performance decrements observed in this study.

## Figures and Tables

**Table 1:** Characteristics of the study participants (n = 14).

Variable	(Mean ± Standard Deviation)
Age (years)	28.00 ± 5.64
Body mass (kg)	82.00 ± 23.93
Experience (years)	3.54 ± 1.11
1RM test (bench press) (kg)	141.43 ± 47.43
1RM test/body mass (kg)	1.82 ± 0.61 *

Legend: 1RM: one repetition maximum. All athletes with loads that keep them in the top 10 of their categories nationwide. * Athletes with values above 1.4 in the bench press (1RM/body weight) would be considered elite athletes [23].

**Table 2:** Skin temperature (mean ± SD; 95% CI) at different times with traditional training and with the use of chains in paralympic powerlifting.

	Pectoral Sternal (°C)	Pectoral Clavicular (°C)	Deltoid Anterior (°C)	Triceps (°C)
Before Traditional “a”	33.29 ± 1.47 (32.44–34.13)	33.76 ± 1.32 (32.99–34.52)	33.80 ± 1.09 (33.17–34.43)	32.01 ± 1.06 (31.40–32.63)
Before Chain “b”	33.36 ± 1.45 (32.52–34.19)	33.79 ± 1.31 (33.03–34.54)	33.79 ± 1.05 (33.18–34.39)	31.93 ± 1.07 (31.31–32.55)
After Traditional “c”	33.73 ± 1.95 (32.60–34.86)	34.47 ± 1.34 (33.69–35.24)	34.18 ± 1.46 (33.34–35.03)	32.81 ± 1.23 (32.09–33.52)
After Chain “d”	34.64 ± 1.95 b (33.52–35.77)	35.79 ± 3.68 b (33.66–37.91)	36.21 ± 3.51 c (34.19–38.24)	33.71 ± 1.54 c (32.82–34.60)
24 h Traditional “e”	33.76 ± 1.74 (32.76–34.76)	34.42 ± 1.31 (33.67–35.18)	34.22 ± 1.28 (33.48–34.96)	32.84 ± 1.65 (31.89–33.80)
24 h Chain “f”	34.36 ± 1.34 (33.59–35.13)	34.79 ± 1.05 (34.18–35.39)	34.64 ± 1.22 (33.94–35.34)	33.50 ± 1.61 e (32.57–34.43)
48 h Traditional “g”	33.53 ± 1.34 (32.75–34.31)	33.89 ± 1.01 (33.31–34.47)	33.68 ± 1.00 (33.10–34.25)	32.26 ± 1.09 (31.63–32.88)
48 h Chain “h”	34.36 ± 1.69 g (33.38–35.33)	34.36 ± 1.55 (33.46–35.25)	34.50 ± 1.61 g (33.57–35.43)	33.00 ± 1.18 g (32.32–33.68)
p	b *p* = 0.040 g *p* = 0.018	b *p* = 0.003	c *p* = 0.026 g *p* = 0.017	c *p* = 0.005 e *p* = 0.045 g *p* = 0.005
F	b = 2.212 * g = 11.966 #	b = 4.495 *	c, g = 17.181 #	c, e, g = 17.781 #
η^2^_p_	b = 0.145 g = 0.479	b = 0.257	c, g = 0.569	c, e, g = 0.578

*p* ≤ 0.05 (ANOVA two-way and post hoc Bonferroni). η^2^_p_ = partial eta squared (small effect ≤ 0.05, medium effect 0.05 to 0.25, high effect 0.25 to 050 and very high effect >050). * Intraclass, # interclass. The letters in the left column represent each condition and moment. In the other columns, the reference letters represent the conditions or moments that presented statistical differences.

## Data Availability

The data that support this study can be obtained at https://www.ufs.br/ Department of Physical Education, accessed on 11 January 2024, or the data will be made available on request.
